# Small heat-shock proteins and their role in mechanical stress

**DOI:** 10.1007/s12192-020-01095-z

**Published:** 2020-04-06

**Authors:** Miranda P. Collier, Justin L.P. Benesch

**Affiliations:** 1 Present Address: Department of Biology, Stanford University, 318 Campus Drive, Stanford, CA 94305 USA; 2 Department of Chemistry, Chemistry Research Laboratory, University of Oxford, Mansfield Road, Oxford, OX1 3TA UK

**Keywords:** Small heat-shock proteins, sHSPs, Molecular chaperones, Proteostasis, Mechanosensing, Mechanical stress, HspB8, Filamin C, FLNC, Monodispersity, Polydispersity, Muscle, Cardiomyocytes

## Abstract

The ability of cells to respond to stress is central to health. Stress can damage folded proteins, which are vulnerable to even minor changes in cellular conditions. To maintain proteostasis, cells have developed an intricate network in which molecular chaperones are key players. The small heat-shock proteins (sHSPs) are a widespread family of molecular chaperones, and some sHSPs are prominent in muscle, where cells and proteins must withstand high levels of applied force. sHSPs have long been thought to act as general interceptors of protein aggregation. However, evidence is accumulating that points to a more specific role for sHSPs in protecting proteins from mechanical stress. Here, we briefly introduce the sHSPs and outline the evidence for their role in responses to mechanical stress. We suggest that sHSPs interact with mechanosensitive proteins to regulate physiological extension and contraction cycles. It is likely that further study of these interactions – enabled by the development of experimental methodologies that allow protein contacts to be studied under the application of mechanical force – will expand our understanding of the activity and functions of sHSPs, and of the roles played by chaperones in general.

## Introduction

**Fig. 1:**
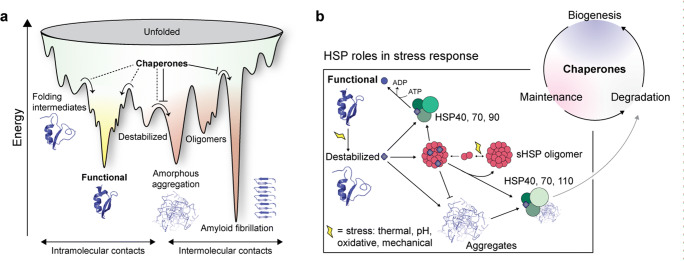
Chaperones modulate the stability of the proteome toward a variety of stressors. **a** A protein energy landscape. Unfolded proteins sample conformations to reach a flexible functional or `native’ state. Destabilized proteins can attract intermolecular interactors through exposure of hydrophobic regions, prompting a cascade to aggregation which can be either protective (Sontag et al. 2017) or pathological (Hipp et al. 2014). Some oligomeric precursors can also form highly thermodynamically stable fibrillar aggregates, which are associated with diseases (Chiti and Dobson 2006). Chaperones affect various pathways along this energy landscape. **b.** The most prominent roles of mammalian heat-shock proteins during cellular response to stress, as part of the maintenance stage of the protein `life cycle’. An environmental, biochemical, or mechanical change causes native protein to misfold. It is then either bound by ATP-dependent HSP machinery (HSP40, HSP70, and HSP90) for refolding or sequestered by sHSPs to prevent aggregation rapidly with less metabolic cost to the cell. Most sHSPs form large oligomers often disrupted by stress conditions (Haslbeck et al. 2016). Additional chaperone complexes (involving HSP40, HSP70, and HSP110) promote aggregate clearance through disassembly or degradation. HSP60 (not pictured) is a mitochondrial chaperone. Schematics influenced by (Kim et al. 2013; Carver et al. 2018; Richter et al. 2010; Garrido et al. 2012)

The ability of cells to respond to stress is central to the health and lifespan of organisms (Morimoto and Cuervo 2014). Various types of stress are particularly damaging to folded proteins, which are only marginally thermodynamically stable in their functioning environments, making them vulnerable to even minor changes to cellular conditions (Kim et al. 2013; Hipp et al. 2014). An increasingly unfolded proteome is functionally impaired and at risk of forming aggregates or amyloid fibrils. If the cell does not manage to degrade or sequester these protein deposits in a controlled manner (Miller et al. 2015; Sontag et al. 2017), they can become pathological (Fig. 1a) (Kakkar et al. 2014; Henning and Brundel 2017; Chiti and Dobson 2006).

To support the integrity of the proteome throughout the protein turnover cycle, from the point of synthesis on the ribosome until degradation, cells across the kingdom of life have developed an intricate network responsible for proteostasis (protein homeostasis, or protein quality control). Comprising an integral part of this network is a family of proteins known as molecular chaperones (Kim et al. 2013; Bukau et al. 2006).

Molecular chaperones can be broadly grouped into two non-exclusive categories: those that assist in de novo folding, and those that sense and mitigate the effects of misfolding at a later stage, performing what has been termed conformational maintenance (Fig. 1b) (Kim et al. 2013). Many of the latter are heat-shock proteins (HSPs), discovered upon their upregulation following thermal stress (Richter et al. 2010; Lindquist 1986). Although the name points exclusively to stress-related function, many human HSPs are constitutively expressed under basal conditions (Labbadia and Morimoto 2015). They are classified by approximate subunit molecular masses into HSP110, HSP90, HSP70, HSP60, HSP40, and the small HSP (sHSP; 16–27 kDa) family.

The paradigmatic sHSP populates oligomers hundreds of kDa in mass which act as a chaperone reservoir. Upon stress, subunits are released, which then tightly sequester misfolded species in a competent state for downstream refolding or degradation to prevent protein aggregation (Fig. 1b) (Haslbeck et al. 2005; Hilton et al. 2013; Garrido et al. 2012). In this chapter, we will dissect this canonical view. After providing a more comprehensive introduction to sHSP function and structure, we will review evidence that challenges the idea that the main function of sHSPs is to broadly sequester misfolded species, suggesting rather that sHSPs may also have specialized roles in responding to mechanical stress. We suggest that further study of sHSP interactions with mechanosensitive clients may elucidate this role and inform a more comprehensive view of the functional landscape of sHSPs and molecular chaperones more broadly.

### Small heat-shock proteins

sHSPs are found in archaea, bacteria, and eukarya, meaning they arose before life diverged, at least 3.5 billion years ago. Their evolutionary trajectory since hints at extensive functional diversity (Basha et al. 2012; Waters 2013). Prokaryotes typically contain very few sHSPs, while most eukaryotes have significantly more – the human genome encodes 10; teleost fish, 13; *C. elegans*, 16; and some land plant species have more than 30 (Marvin et al. 2008; Haslbeck et al. 2005; Waters 2013).

Vertebrate sHSP expression varies with tissue, stage of development, and level of stress (Klemenz et al. 1993; Lutsch et al. 1997; Doran et al. 2007). They are generally abundant, constituting up to 40% of soluble protein in the eye lens (Horwitz et al. 1999) and up to 3% in non-lenticular tissues in the absence of stress (Klemenz et al. 1993; Kato et al. 1991; Dimauro et al. 2017). Some sHSPs serve as a first line of defense when the proteome is compromised by acting as holdases to triage aggregation-prone species (Hilton et al. 2013).

Despite the penetrance of this model of sHSPs as generalist chaperones, the description does not faithfully capture the entire protein family (Basha et al. 2012; McHaourab et al. 2009; Vos et al. 2009). Humans express 10 sHSPs (and the related protein HspB11), categorized based on the presence of a conserved domain (Kappe et al. 2010). Fewer than half of these are upregulated at the onset of stress (The Big Book on Small Heat Shock Proteins 2015), and fewer than half can suppress the aggregation of a broad range of model substrates in vitro, with the rest displaying highly substrate-dependent activity (Table 1) (Mymrikov et al. 2017).

**Table 1:** Features of human sHSPs

Original name	New nomenclature	MW^a^ (kDa)	Tissue distribution^b^	Stress-inducible?^c^	Promiscuous chaperone?^d^
Hsp27	HspB1	22.8	Ubiquitous	Yes	Yes
MKBP	HspB2	20.2	Muscle	No	No
HspL27	HspB3	17.0	Muscle	No	No
αA-crystallin	HspB4	19.9	Eye lens	No	Yes
αB-crystallin	HspB5	20.1	Ubiquitous	Yes	Yes
Hsp20	HspB6	17.1	Muscle, brain	No	No
cvHsp	HspB7	18.6	Muscle	No	No
Hsp22	HspB8	21.6	Muscle, brain	Somewhat	No
CT51	HspB9	17.5	Testis	No	No
ODF1	HspB10	28.4	Testis	No	No

^a^ Molecular weight of the monomer, based on sequences in the UniProt database

^b^ Collated from reviews (The Big Book on Small Heat-Shock Proteins, Chapter 1, 2015 and Garrido et al. 2012) and mRNA levels (Vos et al. 2009)

^c^ From The Big Book of Small Heat-Shock Proteins, Chapter 1.

^d^ From Mymrikov et al. (2017), where Yes denotes significant ability to suppress aggregation in 6 of 6 model substrates

### Functions and interactors

Evidence suggests that in vitro substrate dependence reflects in vivo specificity. sHSP interactomes, though difficult to determine comprehensively, contain proteins that are shared between multiple members of the family and others that are tied to a single sHSP (Mymrikov et al. 2017; Arrigo and Gibert 2013; Arrigo 2013). sHSPs therefore appear to act within a two-tiered specialist-generalist framework, with a majority seemingly adopting specialist functions under physiological conditions. It is as yet unclear whether specialists evolved from generalists, or vice versa. However, it is clear that sHSPs from both types are implicated in human diseases – alterations to certain genes or expression levels are associated with myopathies, neuropathies, cataract, neurodegenerative disease, and cancer (Kakkar et al. 2014; Bakthisaran et al. 2015; Treweek et al. 2015). sHSP-targeted therapeutics are a potential treatment avenue (Salinthone et al. 2008; Henning and Brundel 2017), and their development requires in-depth analyses of the interactions underlying these phenotypes. Thus, an improved understanding of the specialist role of the sHSPs – and with it, their interactors – is potentially of biomedical as well as purely biological interest.

Terminology has been proposed to distinguish between sHSP interactors that are destabilized under stress and those that are bound physiologically by referring to these as substrates and clients, respectively (Strauch and Haslbeck 2016), although the boundaries between the two are not always clear. In muscle for example, which expresses the greatest variety of sHSPs in humans (Table 1), several sHSPs interact with cytoskeletal components and associated proteins (Mounier and Arrigo 2002; Bennardini et al. 1992; Houck and Clark 2010; Wu et al. 2017; Tessier et al. 2003). In striated muscle, many also colocalize with the sarcomere, the basic unit of muscle contraction (Mercer et al. 2018; Golenhofen et al. 2004). These interactions have been reported under basal conditions as well as following oxidative, thermal, and mechanical stress (Koh and Escobedo 2004; Pivovarova et al. 2007; Golenhofen et al. 1999; Shimizu et al. 2016; Ke et al. 2011). They are not yet understood with enough clarity at the molecular level to know whether the sHSPs are targeting native-like proteins in a client-based mechanism, or misfolding proteins in a substrate-based mechanism (Seit-Nebi et al. 2013). Whichever the case may be, these interactions play critical roles in the maintenance of cytoskeletal and muscle structural integrity (Wettstein et al. 2012; Liao et al. 2017; Dreiza et al. 2010), with disruption leading to cardiac and skeletal myopathies (Henning and Brundel 2017; Inagaki et al. 2006; Kumarapeli et al. 2010; Juo et al. 2016; Unger et al. 2017).

Another prominent class of in vivo sHSP interactors is other sHSPs themselves. Dynamic oligomerization is a common feature of the protein family (Fig. 1b). While most oligomeric proteins related by gene duplication do not co-assemble (Hochberg et al. 2018); hetero-oligomerization is common among human sHSPs (Mymrikov et al. 2012; Arrigo 2013; Bakthisaran et al. 2015; Fontaine et al. 2005). This implies an evolutionary constraint in the form of shared function, meaning not only do individual sHSPs perform specialist roles, but the complexes they form may do so as well. The hypothesis is bolstered by tightly balanced constitutive tissue co-expression profiles (Vos et al. 2009; Sugiyama et al. 2000). Disease-linked mutations have been observed to affect heteromerization (Weeks et al. 2018; Simon et al. 2013; Morelli et al. 2017), but this area remains largely unexplored. Elucidating the functional purpose and biophysical determinants of co-assembly is a key aim for the sHSP field.

### Dynamic structure

Structural characterization of sHSPs has proven challenging due to their high degree of plasticity at several levels of protein organization. At the quaternary level, many sHSPs can populate multiple stoichiometries at once (McHaourab et al. 2009; Basha et al. 2012). This is commonly termed “polydispersity,” as opposed to “monodispersity,” where a protein adopts a single quaternary organization. Here, “more” or “less” polydisperse will refer to the relative breadth of stoichiometric distributions.

sHSP polydispersity is dynamic, involving the continual recycling of subunits (Aquilina et al. 2003; Bova et al. 2000). It is also tuneable: in non-metazoa and plants, they are usually monodisperse under non-stressed conditions and become polydisperse with heat stress (The Big Book on Small Heat Shock Proteins 2015). This has enabled structural characterization of several sHSP oligomers by X-ray crystallography from yeast, wheat, archaea, and bacteria (Strauch and Haslbeck 2016). These structures reveal polyhedral or stacked-ring arrangements composed of dimeric building blocks.

Stress-inducible human sHSPs are highly polydisperse under basal conditions. HspB5, for example, forms oligomers ranging from 10 to almost 50 subunits (Hochberg and Benesch 2014). Consequently, these have not yet been fully structurally characterized at high resolution, since their plasticity hampers many biophysical techniques that require homogeneity or report on ensemble averages (Basha et al. 2012). Truncated, and as a result less polydisperse, forms of the proteins have yielded several partial X-ray structures of dimers. HspB6 does not assemble beyond a homo-dimer in its full-length form, and is the only human sHSP that has been crystallized without truncation (Sluchanko et al. 2017). The corresponding structure, in complex with a binding partner, is missing approximately a third of each subunit chain due to high flexibility, making it very similar to truncated structures of other members of the family (Sluchanko et al. 2017). The quaternary organization of vertebrate sHSPs is susceptible to change not just upon heat stress, but also posttranslational modification (PTM), which is very rarely observed in sHSPs across plants, bacteria, archaea, or fungi (Garrido et al. 2012).

**Fig. 2:**
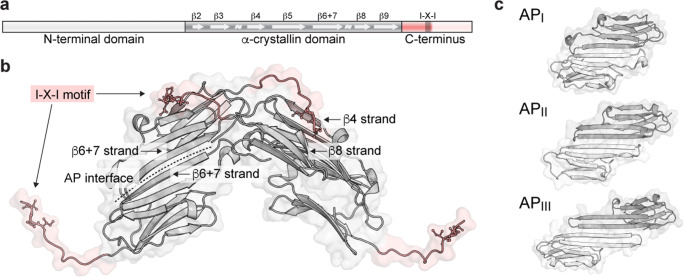
Structural regions of sHSPs and hierarchy of assembly. **a**. The primary sequence encodes three domains: **a** central β-sheet rich ACD flanked by N- and C-terminal regions. Truncating the termini (faded regions) reduces polydispersity and facilitates crystallization. **b**. X-ray structure of the HspB5 ACD and partial C-terminus, showing 4 monomers (Laganowsky et al. 2010). The ACD dimerizes via an antiparallel (AP) interface between strands β6 + 7. The C-terminus bridges dimers by docking into a groove between strands β4 and β8 via the I-X-I motif (sticks; in HspB5, X = proline). **c**. The contacts comprising the AP interface may shift under different conditions, evidenced by the observation of three registers of the HspB5 dimer by X-ray crystallography (PDB IDs 3L1G, 2WJ7, 4M5S). Multiple registers of the HspB1 ACD dimer have also been captured in X-ray structures (H. Gastall, unpublished)

sHSPs assemble via a hierarchy of oligomeric interfaces (Fig. 2a). The α-crystallin domain (ACD), which is highly conserved and defines the family, is located in the middle of the primary sequence and adopts a relatively stable β-strand-rich tertiary structure (Hilton and Benesch 2012). The sHSP dimer, the basic unit of assembly, forms via β-strand pairing within the ACD. β-strands are conventionally numbered, with metazoan sHSPs dimerizing via a combined and extended β6 + 7 -strand in an antiparallel (AP) arrangement in all structures observed to date (Fig. 2a) (Haslbeck et al. 2016; Treweek et al. 2015). This interface is not especially rigid; in the case of HspB5, the ACD-ACD binding affinity is in the low micromolar range, and several distinct registers have been observed in X-ray structures (AP_I_, AP_II_, AP_III_) (Fig. 2b) (Hochberg et al. 2014).

The ACD is flanked by a disordered N-terminal domain (NTD) and a shorter but similarly flexible C-terminal region. The C-terminus is critical for higher order oligomerization. The shortest C-termini are found in the least polydisperse members of the family (Weeks et al. 2014; Boelens et al. 1998); longer C-termini bridge ACD dimers by binding a groove between strands β4 and β8 via a conserved motif, which consists of three residues I-X-I (in some sHSPs, valine replaces one or both isoleucine) (Fig. 2a) (Hilton and Benesch 2012). Finally, inter-subunit interactions involving the NTD with another NTD or ACD have been observed (Jehle et al. 2011; McDonald et al. 2012). These are particularly affected by phosphorylation, which seems to occur exclusively within this domain (Garrido et al. 2012; Heirbaut et al. 2017).

Variability across the three regions of human sHSPs, but particularly the termini (Kriehuber et al. 2010), is believed to underlie differences in target recognition both directly through sequence motifs, and indirectly through modulation of quaternary structure and dynamics. Although there is still much to learn about their mechanisms of action, a clear portrait has emerged of a sHSP system that is finely tuned to aid in addressing the cellular need to maintain a healthy proteome. Constitutive expression, broad interactomes, and rapid mechanisms of energy-independent responsiveness suggest a range of functions from physiological to pathological conditions. Their complexity of structure and function poses many experimental challenges; thus, methods that can tolerate heterogeneity are particularly useful for disentangling sHSP behavior.

### A role for sHSPs in mechanical stress

Two main lines of evidence support the idea that sHSPs may play a role in cellular responses to mechanical stress. The first is indirect: patterns of sHSP expression and subcellular localization suggest biomechanical relevance. sHSPs have been found to colocalize with and bind to components of the cytoskeleton and other proteins involved in muscle function and responses to mechanical stress. sHSP expression has also been found to be relatively high in cells that populate particularly stiff (e.g., spinal cord, skeletal muscle) or continually moving (e.g., heart, diaphragm, developing tissues) microenvironments. The reasons for these colocalization and expression patterns have been presumed to relate to the sHSPs’ role in preventing protein aggregation. The second line of evidence comprises direct links between sHSP functions and pathways involved in sensing and responding to mechanical cues. Building on prior reports of colocalization with direct observation of a sHSP in a stress-response role, our laboratory and collaborators confirmed the interaction of a sHSP (HspB1) with a protein important in musculoskeletal mechanosensing – the actin-binding protein filamin C (FLNC). We elucidated molecular determinants of this sHSP-client interaction and showed that both proteins are upregulated in the context of both acute and chronic biomechanical stress in heart tissue. Below, we briefly highlight evidence supporting sHSP action as mechanical stress sensors, and then summarize our findings on the HspB1-FLNC interaction.

### Evidence for sHSPs as mechanoresponsive chaperones

sHSPs affect muscle contraction and elasticity and colocalize with various proteins that are key components of the cytoskeleton, including actin, titin, and intermediate filament proteins. These interactions have been shown to increase following mechanical loading on cells or tissues, often with accompanying modification of the sHSPs.

HspB1 and HspB5 colocalize with various types of intermediate filaments. HspB1 associates with vimentin (Lee et al. 2005). Vimentin filaments are highly elastic, and can withstand larger deformations than actin and microtubules, which helps them protect the nucleus during cell migration (Patteson et al. 2019). Whether HspB1 binds dynamic vimentin and affects its capacity for mechanical protection is unknown, and would be of interest to investigate. HspB1 also associates with keratin, which remodels extensively in response to routine mechanical stress experienced by the outer epidermal layer (Kayser et al. 2013).

HspB5, and to a lesser degree HspB1, associates with glial fibrillary acidic protein (GFAP), the major intermediate filament protein in astrocytes. GFAP is important for cell shape, strength, and motility; and is expressed at lower levels in astrocytes in the brain compared to higher levels in spinal cord astrocytes, which need to be stiffer (Gorter et al. 2018). HspB5 also associates with desmin, an intermediate filament protein specific to striated muscle. HspB5 mutation R120G is linked to hereditary skeletal muscle desminopathy, which can also be caused by mutations in the *DES* gene. The disease features large desmin-containing aggregates in muscle tissue that are positive for HspB5 and HspB1 (Clemen et al. 2013).

Many sHSPs have been implicated in actin binding and in modulation of the dynamics of actin polymerization into microfilaments. Reports of this aspect of sHSP activity have at times conflicted with one another, and our understanding continues to be refined. HspB1 affects actin polymerization in vitro in a phosphorylation-dependent manner, pointing to a direct interaction (Mounier and Arrigo 2002). Mechanically stressing fibroblasts through cyclical stretch activates p38 MAPK signaling, leading to HspB1 phosphorylation and its recruitment to actin structures at the sites of highest traction force (Hoffman et al. 2017). Similar observations of recruitment to actin fibers have been reported for HspB5, through testing the effects of heat stress (Singh et al. 2007; Yin et al. 2019). Both HspB1 and HspB5 associate with myotube-specific actin bundles (Sugiyama et al. 2000).

HspB6, conversely, was at one time believed to bind actin but has since been postulated to instead affect the cytoskeleton indirectly via its interaction with 14–3-3 proteins, particularly in smooth muscle (Seit-Nebi and Gusev 2010). HspB8 also exerts indirect effects on actin structures in conjunction with its binding partner BAG3, as discussed in more detail below. Most recently, an actin-related role has emerged for HspB7: cardiac-specific HSPB7 KO is lethal in mouse embryos, with abnormally long actin filaments and aberrant bundles leading to disorganized sarcomeres (Wu et al. 2017). Loss of HspB7 also leads to upregulation of Lmod2, an actin-nucleating protein that contributes to cardiomyocyte force generation (Wu et al. 2017). HspB7 may therefore interact with Lmod2, or be able to partially compensate for it in somehow surveilling the initial assembly of actin-based structures under force. The molecular features of sHSP interactions with actin and macromolecular actin-based structures, and how they are altered by the forces exerted on actin filaments in vivo, will be an interesting area of future work.

In addition to sarcomeric actin filaments, the large, elastic sarcomeric protein titin is known to be a target of HspB1 and HspB5. These associations were first confirmed following ischemic stress (Golenhofen et al. 2002), and later mechanical perturbation by subjecting mouse skeletal muscle to cycles of extension and contraction (Koh and Escobedo 2004). Titin binding by HspB1 and HspB5 has since been confirmed in patient samples exhibiting a variety of skeletal muscle myopathies (Unger et al. 2017). While studies have tended to focus on the translocation of sHSPs to titin following major stress, HspB5 in particular also affects the mechanical properties of recombinant titin segments in vitro that have not undergone prior conformational disruption (Bullard et al. 2004; Zhu et al. 2009). As for actin, structural characterization of sHSP-titin interactions under different force regimes will be informative to better understand these processes.

### Tension-related functions of sHSPs

Beyond expression profiles and binding partners, observations that sHSPs can directly alter cellular functions involving biomechanical force transduction and regulation – such as cell adhesion and mitotic spindle alignment – strengthen the case for their involvement in responses to mechanical stress. In the context of cellular adhesion, a reduction in HspB1 in tumoral breast cells or of HspB5 in glioma cells led to reduced cell adhesiveness, as well as other effects downstream of cytoskeletal rearrangement such as altered morphology (Loones et al. 2000). The importance of HspB5 for cell adhesion was reinforced in a more recent study, which emphasized that stress was not a prerequisite for this function. HspB5 knockdown causes both glial and myoblast cells to migrate faster in 2D culture as a result of being less adherent. The phenotype is sensitive to actin and tubulin depolymerization, and features changes in the position and possibly the turnover of vinculin, a critical protein linking cell adhesion complexes to actin stress fibers (Shimizu et al. 2016). Thus future work will delineate the direct and downstream mechanisms by which HspB5 affects adhesiveness, and how these may overlap with HspB1, which has a similar effect on the migration of NIH3T3 fibroblasts (Lee et al. 2008). These mechanisms may also yield insight into the unclear physiological roles of sHSPs in development (Dubinska-Magiera et al. 2014); a period when proper cell migration and positioning is heavily guided by mechanotransduction cues (Haack and Abdelilah-Seyfried 2016).

HspB5 also safeguards cardiomyocytes through a direct interaction with focal adhesion kinase (FAK). FAK mediates the stretch response and protects against apoptosis in cardiomyocytes, but it can be cleaved by calpains and lose this function. HspB5 binds FAK in a cell-stretch-dependent manner, shielding it from proteolysis; the HspB5-FAK interaction was barely detected in the absence of mechanical stress. Levels of FAK remained the same before and after the cellular stretch response, which resulted in HspB5 phosphorylation. These effects were validated in vivo by analysis of the hearts of transgenic mice. Altogether, these findings point to a mechanosensitive mechanism for HspB5 at the molecular scale (Pereira et al. 2014).

Mechanotransduction occurs in widespread cellular processes. During cell division, force sensing is required for the alignment of mitotic spindles; spindles will self-assemble in a cell extract, but without environmental cues, the orientation is random. Fuchs et al. (2015) showed that the HspB8/BAG3 complex aids in the process of transducing the cues to position the spindle, with depletion of HspB8 (or BAG3 or p62) resulting in disorganized chromosome retraction fibers, which normally exert pulling forces on the spindle. Biochemical stiffening of the mitotic actin cortex rescued the phenotype.

Whereas its function in spindle alignment was described in HeLa cells and may occur in a variety of cell types, the HspB8/BAG3 complex also plays a specific role in muscle tissue. In concert with Hsp70, HspB8 and BAG3 are required for the only known autophagic pathway induced by external tension, termed chaperone-assisted selective autophagy (CASA) (Arndt et al. 2010). CASA helps to maintain the integrity of the sarcomeric Z-disk by recognizing mechanically damaged filamin, an actin-binding protein, and releasing it from the sarcomere for degradation (Ulbricht et al. 2013). BAG3 performs a range of functions in protein quality control, through interactions with multiple proteostasis factors including Hsp70 chaperones, ubiquitin ligases, autophagy receptors, and trafficking cargo (Klimek et al. 2017). Any involvement of BAG3 in force-mediated quality control pathways described to date, however, requires HspB8.

### HspB1 interacts with filamin C in a phosphorylation-dependent and mechanosensitive manner

As discussed above, the sHSPs are frequently associated with both cytoskeletal proteins (Robinson et al. 2010; Snoeckx et al. 2001) and with other cellular machinery that enables muscle contraction (Golenhofen et al. 2004), suggesting that sHSP-client interactions may play a role in mechanosensitivity. To test this hypothesis, we conducted a detailed analysis of an sHSP-client pair – HspB1 and filamin C (FLNC) – that was previously reported to potentially interact in vivo.

Filamins are involved in cellular signaling, motility and differentiation, and cytoskeletal organization (Razinia et al. 2012). Human filamins exist as homodimers with a molecular weight of about 280 kDa, with each monomer consisting of an actin-binding domain at the N-terminal and 24 immunoglobulin (Ig)-like domains. FLNC is found in striated muscle and associates with thin filaments of sarcomeric actin (van der Ven et al. 2000). Alterations of the FLNC gene have been linked in humans to skeletal myopathies, and to cardiac abnormalities and pathologies (Furst et al. 2013; Ortiz-Genga et al. 2016; Brodehl et al. 2016). There is evidence that FLNC can sense local force (Sutherland-Smith 2011; Lad et al. 2008; Fujita et al. 2012), though the mechanism by which it does so is not yet fully elucidated.

HspB1 is also generally highly expressed in striated muscle. Results from a yeast two-hybrid assay first indicated that HspB1 may interact with FLNC (van der Ven et al. 2006). HspB1 has also been found to be prevalent in protein aggregates collected from patients with skeletal myopathies caused by FLNC mutations (Kley et al. 2013) and colocalizes at sarcomeric lesions with FLNC (Chevessier et al. 2015), suggesting that it may associate with FLNC during stress. Based on reports that HspB1 is phosphorylated in response to mechanical cues in cells and tissue, we also had reason to suspect this modification may be important for modulating the interaction of HspB1 with mechanosensitive clients such as FLNC. Phosphorylated HspB1 translocates to the sarcomeric Z-discs in striated muscle (Koh and Escobedo 2004; Hu et al. 2017) and to tension-bearing cytoskeletal fibers in fibroblasts (Hoffman et al. 2017).

**Fig. 3:**
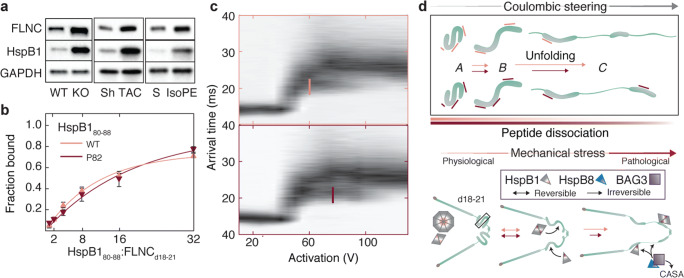
Phosphorylated HspB1 modulates the extension of FLNC with implications for cardiac function under mechanical stress. **a**. Western blots of FLNC, HspB1, and GAPDH as loading control from mouse hearts reveal upregulation of both proteins following mechanical stress. WT = wild-type; KO = muscle LIM protein knockout, a transgenic model of biomechanical dysfunction; Sh = sham surgery control; TAC = transverse aortic constriction; S = saline control; IsoPE = isoprenaline/epinephrine treatment. **b**. Measurement of FLNC domains 18–21 binding to peptides derived from HspB1 residues 80–88, without and with phosphorylation at Ser82. **c**. Coulombically steered unfolding of FLNC domains 18–21 bound to a single HspB1 peptide, unmodified (top) or phosphorylated (bottom). Lines designate the activation required to transition half of an intermediate FLNC state to a more unfolded state, which is delayed when bound to HspB1 phosphopeptide. **d**. Schematic of force-induced changes to FLNC captured by coulombic unfolding, in relation to full-length FLNC, HspB1 peptide binding, and HspB8/BAG3 mediated clearance. This figure is derived from Collier et al. 2019 (DOI: 10.1126/sciadv.aav8421), licensed under CC BY 4.0

We explored in more depth the interaction between HspB1 and FLNC, and the role that HspB1 phosphorylation plays in modulating that interaction (Collier et al. 2019). We confirmed, using immunoblotting and immunoprecipitation, that HspB1 and FLNC are upregulated and interact in mouse hearts subjected to biomechanical stress (Collier et al. 2019). This upregulation was consistently observed in hearts subjected to three different models of biomechanical stress (disease, chemical treatment and mechanical treatment) (Fig. 3a). We also found that HspB1 undergoes phosphorylation in the stressed heart, and that this results in structural rearrangements within HspB1 in vitro that make its FLNC-binding region more flexible and thus accessible (Collier et al. 2019).

To recreate experimentally the mechanical forces FLNC undergoes in cells, we applied a coulombic force-unfolding approach where mass-selected molecules are extended, isolated in vacuum. The unfolding experiment allowed us to observe how the phosphorylated region of HspB1 affects FLNC extension, despite phosphorylation having no measurable effect on affinity to FLNC without extension (Fig. 3b). We found that HspB1 phosphorylation inhibited a partially unfolded form of FLNC from unfolding further, potentially protecting it from over-extension during mechanical stress. Phosphorylated HspB1 peptide bound FLNC and modulated its unfolding while the same peptide, when unphosphorylated, bound FLNC but had no effect on unfolding (Fig. 3c). The phosphorylated peptide also remained bound to FLNC domains longer through their unfolding trajectory than when unphosphorylated (Fig. 3d).

Altogether, our results demonstrate that HspB1 has an important role in regulating how FLNC responds to mechanical stress – a role that goes beyond preventing aggregation of an already misfolding population of a protein. Further questions to explore in the context of the HspB1-FLNC interaction include whether the interaction depends on the rate of force loading on FLNC, and how HspB1 phosphorylation may affect FLNC stability and turnover in vivo. It has also been reported that HspB7 binds to FLNC, though at a different site (Juo et al. 2016); this raises the question of why multiple sHSPs would be targeted to a mechanosensing protein, and whether hetero-oligomerization plays a role. Finally, it will be informative to explore how the “molecular decision” is made whether to protect FLNC with HspB1 or target it for degradation via chaperone-assisted selective autophagy involving the HspB8/BAG3 chaperone complex.

## Conclusion

In this chapter, we have presented an overview of the sHSPs, including their function, interactors and dynamic structure. We have also outlined evidence of specialist, rather than generalist, functions for sHSPs in responding to mechanical stress. This evidence encompasses over two decades of reports that sHSPs colocalize with proteins involved in bearing and transducing mechanical cues, and that sHSPs are functionally implicated in mechanical stress responses. We have also summarized the recent discovery of phosphorylation and upregulation of HspB1 in the hearts of a mouse model of heart failure alongside an interaction with FLNC, pointing to a force-dependent mode of strengthened client binding upon HspB1 phosphorylation.

### A proposal for force-focused proteostasis research

Overall, we do not believe that the totality of sHSP functions in mechanical stress can be entirely explained by prevention of protein aggregation or by modulating the kinetics of cytoskeletal (de)polymerization. In our view, emerging findings indicate that further study of sHSPs with putative mechanosensing interactors could be highly revealing. Such exploration, taking advantage of increasingly sensitive experimental techniques that allow protein-protein interactions to be quantified under the application of force, would likely uncover novel mechanisms of protein quality control and conformational surveillance. In addition, cell- and tissue-scale screens seeking sHSP mechanical stress roles, and associated binding partners in refolding and disposal pathways, could uncover instances of specialized activity that have evaded detection without accounting for tension dependence. Lastly, these functions raise the question of whether sHSPs themselves, in addition to their binding partners, access functionally relevant force-dependent conformations. It would be interesting to turn to single molecule techniques, for example, and test whether force magnitudes and rates affect sHSP client binding or the registers and affinities of sHSP oligomeric interfaces. The results could demand that we rethink our understanding of the role and activities of sHSPs and, given reports of other interactions between HSP family proteins and clients unfolded by force (Mashaghi et al. 2016; Perales-Calvo et al. 2018; Simoes-Correia et al. 2014), potentially enlighten our understanding of the specific roles molecular chaperones play in mechanically responsive cells.

### A framework for categorizing sHSP function

**Fig. 4:**
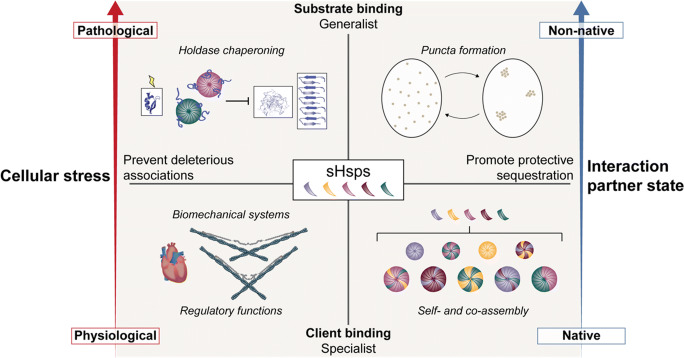
Conceptual view of the sHSP functional landscape

Bringing together the broader literature on the processes involving sHSPs, we conceptualize their function in a framework that encompasses a multitude of roles from physiological to pathological conditions. This landscape can be subdivided coarsely into four quadrants (Fig. 4). Under minimal stress, when most proteins in the cell can be presumed to populate their native states, sHSPs are primarily engaged in specialist client-binding (lower left). Also in the specialist descriptor, we include self- and co-assembly, since these interactions are both native-state and precise (lower right). With mounting stress, the proteome becomes increasingly destabilized. Some sHSPs then adopt generalist roles, sequestering misfolded substrates in contained membraneless inclusions (upper right) or forming soluble complexes with them in order to prevent cascades to amorphous aggregates or amyloid fibrils in canonical holdase fashion (upper left).

Thus, the left and right division arises between functions that serve to prevent interactions that should not occur (left) and ones that facilitate interactions that are protective (right); for example, oligomerization can shield promiscuous interfaces when they are not needed. Certain sHSPs cover more of this landscape than others, and biochemical changes – to the environment or in the form of protein modification – can shift their focus between quadrants. Of particular note are native partner sHSP interactions, which have received little mechanistic attention compared to roles fitting the generalist paradigm. We place biomechanical systems in the preventative-client quadrant, and note that the term `native’ as it applies to these systems must account for the routine structural distortions induced by physiological force, which may bridge a biophysical gap between misfolding substrates and more rigid clients. Studies from our laboratory and others, as well as sHSP abundance in muscle and implications in musculoskeletal pathologies, strongly hint at the existence of other clients that may fit into this piece of an expanded sHSP paradigm.

## References

[CR1] Aquilina JA, Benesch JL, Bateman OA, Slingsby C, Robinson CV (2003). Polydispersity of a mammalian chaperone: mass spectrometry reveals the population of oligomers in alphaB-crystallin. Proc Natl Acad Sci U S A.

[CR2] Arndt V, Dick N, Tawo R, Dreiseidler M, Wenzel D, Hesse M, Furst DO, Saftig P, Saint R, Fleischmann BK, Hoch M, Hohfeld J (2010). Chaperone-assisted selective autophagy is essential for muscle maintenance. Curr Biol.

[CR3] Arrigo AP (2013). Human small heat shock proteins: protein interactomes of homo- and hetero-oligomeric complexes: an update. FEBS Lett.

[CR4] Arrigo AP, Gibert B (2013). Protein interactomes of three stress inducible small heat shock proteins: HspB1, HspB5 and HspB8. Int J Hyperth.

[CR5] Bakthisaran R, Tangirala R, Rao Ch M (2015). Small heat shock proteins: role in cellular functions and pathology. Biochim Biophys Acta.

[CR6] Basha E, O'Neill H, Vierling E (2012). Small heat shock proteins and alpha-crystallins: dynamic proteins with flexible functions. Trends Biochem Sci.

[CR7] Bennardini F, Wrzosek A, Chiesi M (1992). Alpha B-crystallin in cardiac tissue. Association with actin and desmin filaments. Circ Res.

[CR8] The Big Book on Small Heat Shock Proteins (2015) The multicolored world of the human HSPB family. In: Tanguay RM, Hightower L (eds) The big book on small heat shock proteins, Springer International Publishing, Cham, pp 3–26

[CR9] Boelens WC, Van Boekel MAM, De Jong WW (1998). HspB3, the most deviating of the six known human small heat shock proteins. Biochim Biophys Acta, Protein Struct Mol Enzymol.

[CR10] Bova MP, McHaourab HS, Han Y, Fung BK (2000). Subunit exchange of small heat shock proteins. Analysis of oligomer formation of alphaA-crystallin and Hsp27 by fluorescence resonance energy transfer and site-directed truncations. J Biol Chem.

[CR11] Brodehl A, Ferrier RA, Hamilton SJ, Greenway SC, Brundler MA, Yu W, Gibson WT, McKinnon ML, McGillivray B, Alvarez N, Giuffre M, Schwartzentruber J, Gerull B, Consortium FC (2016). Mutations in FLNC are associated with familial restrictive cardiomyopathy. Hum Mutat.

[CR12] Bukau B, Weissman J, Horwich A (2006). Molecular chaperones and protein quality control. Cell.

[CR13] Bullard B, Ferguson C, Minajeva A, Leake MC, Gautel M, Labeit D, Ding L, Labeit S, Horwitz J, Leonard KR, Linke WA (2004). Association of the chaperone alphaB-crystallin with titin in heart muscle. J Biol Chem.

[CR14] Carver JA, Ecroyd H, Truscott RJW, Thorn DC, Holt C (2018). Proteostasis and the regulation of intra- and extracellular protein aggregation by ATP-independent molecular chaperones: Lens alpha-Crystallins and Milk caseins. Acc Chem Res.

[CR15] Chevessier F, Schuld J, Orfanos Z, Plank AC, Wolf L, Maerkens A, Unger A, Schlotzer-Schrehardt U, Kley RA, Von Horsten S, Marcus K, Linke WA, Vorgerd M, van der Ven PF, Furst DO, Schroder R (2015). Myofibrillar instability exacerbated by acute exercise in filaminopathy. Hum Mol Genet.

[CR16] Chiti F, Dobson CM (2006). Protein misfolding, functional amyloid, and human disease. Annu Rev Biochem.

[CR17] Clemen CS, Herrmann H, Strelkov SV, Schroder R (2013). Desminopathies: pathology and mechanisms. Acta Neuropathol.

[CR18] Collier MP, Alderson TR, de Villiers CP, Nicholls D, Gastall HY, Allison TM, Degiacomi MT, Jiang H, Mlynek G, Furst DO, van der Ven PFM, Djinovic-Carugo K, Baldwin AJ, Watkins H, Gehmlich K, Benesch JLP (2019) HspB1 phosphorylation regulates its intramolecular dynamics and mechanosensitive molecular chaperone interaction with filamin C. Sci Adv 5 (5):eaav8421. 10.1126/sciadv.aav8421 10.1126/sciadv.aav8421 PMC6530996 31131323

[CR19] Dimauro I, Antonioni A, Mercatelli N, Caporossi D (2017). The role of alphaB-crystallin in skeletal and cardiac muscle tissues. Cell Stress Chaperones.

[CR20] Doran P, Gannon J, O'Connell K, Ohlendieck K (2007). Aging skeletal muscle shows a drastic increase in the small heat shock proteins alphaB-crystallin/HspB5 and cvHsp/HspB7. Eur J Cell Biol.

[CR21] Dreiza CM, Komalavilas P, Furnish EJ, Flynn CR, Sheller MR, Smoke CC, Lopes LB, Brophy CM (2010). The small heat shock protein, HSPB6, in muscle function and disease. Cell Stress Chaperones.

[CR22] Dubinska-Magiera M, Jablonska J, Saczko J, Kulbacka J, Jagla T, Daczewska M (2014). Contribution of small heat shock proteins to muscle development and function. FEBS Lett.

[CR23] Fontaine JM, Sun X, Benndorf R, Welsh MJ (2005). Interactions of HSP22 (HSPB8) with HSP20, alphaB-crystallin, and HSPB3. Biochem Biophys Res Commun.

[CR24] Fuchs M, Luthold C, Guilbert SM, Varlet AA, Lambert H, Jette A, Elowe S, Landry J, Lavoie JN (2015). A role for the chaperone complex BAG3-HSPB8 in actin dynamics, spindle orientation and proper chromosome segregation during mitosis. PLoS Genet.

[CR25] Fujita M, Mitsuhashi H, Isogai S, Nakata T, Kawakami A, Nonaka I, Noguchi S, Hayashi YK, Nishino I, Kudo A (2012). Filamin C plays an essential role in the maintenance of the structural integrity of cardiac and skeletal muscles, revealed by the medaka mutant zacro. Dev Biol.

[CR26] Furst DO, Goldfarb LG, Kley RA, Vorgerd M, Olive M, van der Ven PF (2013). Filamin C-related myopathies: pathology and mechanisms. Acta Neuropathol.

[CR27] Garrido C, Paul C, Seigneuric R, Kampinga HH (2012). The small heat shock proteins family: the long forgotten chaperones. Int J Biochem Cell Biol.

[CR28] Golenhofen N, Arbeiter A, Koob R, Drenckhahn D (2002). Ischemia-induced association of the stress protein alpha B-crystallin with I-band portion of cardiac titin. J Mol Cell Cardiol.

[CR29] Golenhofen N, Htun P, Ness W, Koob R, Schaper W, Drenckhahn D (1999). Binding of the stress protein alpha B-crystallin to cardiac myofibrils correlates with the degree of myocardial damage during ischemia/reperfusion in vivo. J Mol Cell Cardiol.

[CR30] Golenhofen N, Perng MD, Quinlan RA, Drenckhahn D (2004). Comparison of the small heat shock proteins alphaB-crystallin, MKBP, HSP25, HSP20, and cvHSP in heart and skeletal muscle. Histochem Cell Biol.

[CR31] Gorter RP, Nutma E, Jahrei MC, de Jonge JC, Quinlan RA, van der Valk P, van Noort JM, Baron W, Amor S (2018). Heat shock proteins are differentially expressed in brain and spinal cord: implications for multiple sclerosis. Clin Exp Immunol.

[CR32] Haack T, Abdelilah-Seyfried S (2016). The force within: endocardial development, mechanotransduction and signalling during cardiac morphogenesis. Development.

[CR33] Haslbeck M, Franzmann T, Weinfurtner D, Buchner J (2005). Some like it hot: the structure and function of small heat-shock proteins. Nat Struct Mol Biol.

[CR34] Haslbeck M, Peschek J, Buchner J, Weinkauf S (2016). Structure and function of alpha-crystallins: traversing from in vitro to in vivo. Biochim Biophys Acta.

[CR35] Heirbaut M, Lermyte F, Martin EM, Beelen S, Sobott F, Strelkov SV, Weeks SD (2017). Specific sequences in the N-terminal domain of human small heat-shock protein HSPB6 dictate preferential hetero-oligomerization with the orthologue HSPB1. J Biol Chem.

[CR36] Henning RH, Brundel B (2017). Proteostasis in cardiac health and disease. Nat Rev Cardiol.

[CR37] Hilton GR, Benesch JL (2012). Two decades of studying non-covalent biomolecular assemblies by means of electrospray ionization mass spectrometry. J R Soc Interface.

[CR38] Hilton GR, Lioe H, Stengel F, Baldwin AJ, Benesch JL (2013). Small heat-shock proteins: paramedics of the cell. Top Curr Chem.

[CR39] Hipp MS, Park SH, Hartl FU (2014). Proteostasis impairment in protein-misfolding and -aggregation diseases. Trends Cell Biol.

[CR40] Hochberg GK, Benesch JL (2014). Dynamical structure of alphaB-crystallin. Prog Biophys Mol Biol.

[CR41] Hochberg GK, Ecroyd H, Liu C, Cox D, Cascio D, Sawaya MR, Collier MP, Stroud J, Carver JA, Baldwin AJ, Robinson CV, Eisenberg DS, Benesch JL, Laganowsky A (2014). The structured core domain of alphaB-crystallin can prevent amyloid fibrillation and associated toxicity. Proc Natl Acad Sci U S A.

[CR42] Hochberg GKA, Shepherd DA, Marklund EG, Santhanagoplan I, Degiacomi MT, Laganowsky A, Allison TM, Basha E, Marty MT, Galpin MR, Struwe WB, Baldwin AJ, Vierling E, Benesch JLP (2018). Structural principles that enable oligomeric small heat-shock protein paralogs to evolve distinct functions. Science.

[CR43] Hoffman L, Jensen CC, Yoshigi M, Beckerle M (2017) Mechanical signals activate p38 MAPK pathway-dependent reinforcement of actin via mechanosensitive HspB1. Mol Biol Cell. 10.1091/mbc.E17-02-0087 10.1091/mbc.E17-02-0087 PMC5620374 28768826

[CR44] Horwitz J, Bova MP, Ding LL, Haley DA, Stewart PL (1999). Lens alpha-crystallin: function and structure. Eye (Lond).

[CR45] Houck SA, Clark JI (2010). Dynamic subunit exchange and the regulation of microtubule assembly by the stress response protein human alphaB crystallin. PLoS One.

[CR46] Hu X, Van Marion DMS, Wiersma M, Zhang D, Brundel B (2017). The protective role of small heat shock proteins in cardiac diseases: key role in atrial fibrillation. Cell Stress Chaperones.

[CR47] Inagaki N, Hayashi T, Arimura T, Koga Y, Takahashi M, Shibata H, Teraoka K, Chikamori T, Yamashina A, Kimura A (2006). Alpha B-crystallin mutation in dilated cardiomyopathy. Biochem Biophys Res Commun.

[CR48] Jehle S, Vollmar BS, Bardiaux B, Dove KK, Rajagopal P, Gonen T, Oschkinat H, Klevit RE (2011). N-terminal domain of alphaB-crystallin provides a conformational switch for multimerization and structural heterogeneity. Proc Natl Acad Sci U S A.

[CR49] Juo LY, Liao WC, Shih YL, Yang BY, Liu AB, Yan YT (2016). HSPB7 interacts with dimerized FLNC and its absence results in progressive myopathy in skeletal muscles. J Cell Sci.

[CR50] Kakkar V, Meister-Broekema M, Minoia M, Carra S, Kampinga HH (2014). Barcoding heat shock proteins to human diseases: looking beyond the heat shock response. Dis Model Mech.

[CR51] Kappe G, Boelens WC, de Jong WW (2010). Why proteins without an alpha-crystallin domain should not be included in the human small heat shock protein family HSPB. Cell Stress Chaperones.

[CR52] Kato K, Shinohara H, Kurobe N, Inaguma Y, Shimizu K, Ohshima K (1991). Tissue distribution and developmental profiles of Immunoreactive alpha-B-Crystallin in the rat determined with a sensitive immunoassay system. Biochim Biophys Acta.

[CR53] Kayser J, Haslbeck M, Dempfle L, Krause M, Grashoff C, Buchner J, Herrmann H, Bausch AR (2013). The small heat shock protein Hsp27 affects assembly dynamics and structure of keratin intermediate filament networks. Biophys J.

[CR54] Ke L, Meijering RA, Hoogstra-Berends F, Mackovicova K, Vos MJ, Van Gelder IC, Henning RH, Kampinga HH, Brundel BJ (2011). HSPB1, HSPB6, HSPB7 and HSPB8 protect against RhoA GTPase-induced remodeling in tachypaced atrial myocytes. PLoS One.

[CR55] Kim YE, Hipp MS, Bracher A, Hayer-Hartl M, Hartl FU (2013). Molecular chaperone functions in protein folding and proteostasis. Annu Rev Biochem.

[CR56] Klemenz R, Andres AC, Frohli E, Schafer R, Aoyama A (1993). Expression of the murine small heat-shock proteins Hsp 25 and alpha-B Crystallin in the absence of stress. J Cell Biol.

[CR57] Kley RA, Maerkens A, Leber Y, Theis V, Schreiner A, van der Ven PF, Uszkoreit J, Stephan C, Eulitz S, Euler N, Kirschner J, Muller K, Meyer HE, Tegenthoff M, Furst DO, Vorgerd M, Muller T, Marcus K (2013). A combined laser microdissection and mass spectrometry approach reveals new disease relevant proteins accumulating in aggregates of filaminopathy patients. Mol Cell Proteomics.

[CR58] Klimek C, Kathage B, Wordehoff J, Hohfeld J (2017). BAG3-mediated proteostasis at a glance. J Cell Sci.

[CR59] Koh TJ, Escobedo J (2004). Cytoskeletal disruption and small heat shock protein translocation immediately after lengthening contractions. Am J Phys Cell Phys.

[CR60] Kriehuber T, Rattei T, Weinmaier T, Bepperling A, Haslbeck M, Buchner J (2010). Independent evolution of the core domain and its flanking sequences in small heat shock proteins. FASEB J.

[CR61] Kumarapeli AR, Horak K, Wang X (2010). Protein quality control in protection against systolic overload cardiomyopathy: the long term role of small heat shock proteins. Am J Transl Res.

[CR62] Labbadia J, Morimoto RI (2015). The biology of proteostasis in aging and disease. Annu Rev Biochem.

[CR63] Lad Y, Jiang P, Ruskamo S, Harburger DS, Ylanne J, Campbell ID, Calderwood DA (2008). Structural basis of the migfilin-filamin interaction and competition with integrin beta tails. J Biol Chem.

[CR64] Laganowsky A, Benesch JL, Landau M, Ding L, Sawaya MR, Cascio D, Huang Q, Robinson CV, Horwitz J, Eisenberg D (2010). Crystal structures of truncated alphaA and alphaB crystallins reveal structural mechanisms of polydispersity important for eye lens function. Protein Sci.

[CR65] Lee JS, Zhang MH, Yun EK, Geum D, Kim K, Kim TH, Lim YS, Seo JS (2005). Heat shock protein 27 interacts with vimentin and prevents insolubilization of vimentin subunits induced by cadmium. Exp Mol Med.

[CR66] Lee JW, Kwak HJ, Lee JJ, Kim YN, Lee JW, Park MJ, Jung SE, Hong SI, Lee JH, Lee JS (2008). HSP27 regulates cell adhesion and invasion via modulation of focal adhesion kinase and MMP-2 expression. Eur J Cell Biol.

[CR67] Liao WC, Juo LY, Shih YL, Chen YH, Yan YT (2017). HSPB7 prevents cardiac conduction system defect through maintaining intercalated disc integrity. PLoS Genet.

[CR68] Lindquist S (1986). The heat-shock response. Annu Rev Biochem.

[CR69] Loones MT, Chang Y, Morange M (2000). The distribution of heat shock proteins in the nervous system of the unstressed mouse embryo suggests a role in neuronal and non-neuronal differentiation. Cell Stress Chaperones.

[CR70] Lutsch G, Vetter R, Offhauss U, Wieske M, Grone HJ, Klemenz R, Schimke I, Stahl J, Benndorf R (1997). Abundance and location of the small heat shock proteins HSP25 and alphaB-crystallin in rat and human heart. Circulation.

[CR71] Marvin M, O'Rourke D, Kurihara T, Juliano CE, Harrison KL, Hutson LD (2008). Developmental expression patterns of the zebrafish small heat shock proteins. Dev Dyn.

[CR72] Mashaghi A, Bezrukavnikov S, Minde DP, Wentink AS, Kityk R, Zachmann-Brand B, Mayer MP, Kramer G, Bukau B, Tans SJ (2016). Alternative modes of client binding enable functional plasticity of Hsp70. Nature.

[CR73] McDonald ET, Bortolus M, Koteiche HA, McHaourab HS (2012). Sequence, structure, and dynamic determinants of Hsp27 (HspB1) equilibrium dissociation are encoded by the N-terminal domain. Biochemistry.

[CR74] McHaourab HS, Godar JA, Stewart PL (2009). Structure and mechanism of protein stability sensors: chaperone activity of small heat shock proteins. Biochemistry.

[CR75] Mercer EJ, Lin YF, Cohen-Gould L, Evans T (2018) Hspb7 is a cardioprotective chaperone facilitating sarcomeric proteostasis. Dev Biol. 10.1016/j.ydbio.2018.01.005 10.1016/j.ydbio.2018.01.005 PMC5818303 29331499

[CR76] Miller SB, Mogk A, Bukau B (2015). Spatially organized aggregation of misfolded proteins as cellular stress defense strategy. J Mol Biol.

[CR77] Morelli FF, Verbeek DS, Bertacchini J, Vinet J, Mediani L, Marmiroli S, Cenacchi G, Nasi M, De Biasi S, Brunsting JF, Lammerding J, Pegoraro E, Angelini C, Tupler R, Alberti S, Carra S (2017). Aberrant compartment formation by HSPB2 Mislocalizes Lamin a and compromises nuclear integrity and function. Cell Rep.

[CR78] Morimoto RI, Cuervo AM (2014). Proteostasis and the aging proteome in health and disease. J Gerontol A Biol Sci Med Sci.

[CR79] Mounier N, Arrigo AP (2002). Actin cytoskeleton and small heat shock proteins: how do they interact?. Cell Stress Chaperones.

[CR80] Mymrikov EV, Daake M, Richter B, Haslbeck M, Buchner J (2017). The chaperone activity and substrate Spectrum of human small heat shock proteins. J Biol Chem.

[CR81] Mymrikov EV, Seit-Nebi AS, Gusev NB (2012). Heterooligomeric complexes of human small heat shock proteins. Cell Stress Chaperones.

[CR82] Ortiz-Genga MF, Cuenca S, Dal Ferro M, Zorio E, Salgado-Aranda R, Climent V, Padron-Barthe L, Duro-Aguado I, Jimenez-Jaimez J, Hidalgo-Olivares VM, Garcia-Campo E, Lanzillo C, Suarez-Mier MP, Yonath H, Marcos-Alonso S, Ochoa JP, Santome JL, Garcia-Giustiniani D, Rodriguez-Garrido JL, Dominguez F, Merlo M, Palomino J, Pena ML, Trujillo JP, Martin-Vila A, Stolfo D, Molina P, Lara-Pezzi E, Calvo-Iglesias FE, Nof E, Calo L, Barriales-Villa R, Gimeno-Blanes JR, Arad M, Garcia-Pavia P, Monserrat L (2016). Truncating FLNC mutations are associated with high-risk dilated and Arrhythmogenic cardiomyopathies. J Am Coll Cardiol.

[CR83] Patteson AE, Vahabikashi A, Pogoda K, Adam SA, Mandal K, Kittisopikul M, Sivagurunathan S, Goldman A, Goldman RD, Janmey PA (2019) Vimentin protects cells against nuclear rupture and DNA damage during migration. J Cell Biol. 10.1083/jcb.201902046 10.1083/jcb.201902046 PMC6891099 31676718

[CR84] Perales-Calvo J, Giganti D, Stirnemann G, Garcia-Manyes S (2018) The force-dependent mechanism of DnaK-mediated mechanical folding. Sci Adv 4(2). 10.1126/sciadv.aaq0243 10.1126/sciadv.aaq0243 PMC5817926 29487911

[CR85] Pereira MB, Santos AM, Goncalves DC, Cardoso AC, Consonni SR, Gozzo FC, Oliveira PS, Pereira AH, Figueiredo AR, Tiroli-Cepeda AO, Ramos CH, de Thomaz AA, Cesar CL, Franchini KG (2014). alphaB-crystallin interacts with and prevents stress-activated proteolysis of focal adhesion kinase by calpain in cardiomyocytes. Nat Commun.

[CR86] Pivovarova AV, Chebotareva NA, Chernik IS, Gusev NB, Levitsky DI (2007). Small heat shock protein Hsp27 prevents heat-induced aggregation of F-actin by forming soluble complexes with denatured actin. FEBS J.

[CR87] Razinia Z, Makela T, Ylanne J, Calderwood DA (2012). Filamins in mechanosensing and signaling. Annu Rev Biophys.

[CR88] Richter K, Haslbeck M, Buchner J (2010). The heat shock response: life on the verge of death. Mol Cell.

[CR89] Robinson AA, Dunn MJ, McCormack A, dos Remedios C, Rose ML (2010). Protective effect of phosphorylated Hsp27 in coronary arteries through actin stabilization. J Mol Cell Cardiol.

[CR90] Salinthone S, Tyagi M, Gerthoffer WT (2008). Small heat shock proteins in smooth muscle. Pharmacol Ther.

[CR91] Seit-Nebi AS, Datskevich P, Gusev NB (2013). Commentary on paper: small heat shock proteins and the cytoskeleton: an essential interplay for cell integrity? (Wettstein et al.). Int J Biochem Cell Biol.

[CR92] Seit-Nebi AS, Gusev NB (2010). Versatility of the small heat shock protein HSPB6 (Hsp20). Cell Stress Chaperones.

[CR93] Shimizu M, Tanaka M, Atomi Y (2016). Small heat shock protein alphaB-Crystallin controls shape and adhesion of Glioma and myoblast cells in the absence of stress. PLoS One.

[CR94] Simoes-Correia J, Silva DI, Melo S, Figueiredo J, Caldeira J, Pinto MT, Girao H, Pereira P, Seruca R (2014). DNAJB4 molecular chaperone distinguishes WT from mutant E-cadherin, determining their fate in vitro and in vivo. Hum Mol Genet.

[CR95] Simon S, Dimitrova V, Gibert B, Virot S, Mounier N, Nivon M, Kretz-Remy C, Corset V, Mehlen P, Arrigo AP (2013). Analysis of the dominant effects mediated by wild type or R120G mutant of alphaB-crystallin (HspB5) towards Hsp27 (HspB1). PLoS One.

[CR96] Singh BN, Rao KS, Ramakrishna T, Rangaraj N, Rao Ch M (2007). Association of alphaB-crystallin, a small heat shock protein, with actin: role in modulating actin filament dynamics in vivo. J Mol Biol.

[CR97] Sluchanko NN, Beelen S, Kulikova AA, Weeks SD, Antson AA, Gusev NB, Strelkov SV (2017). Structural basis for the interaction of a human small heat shock protein with the 14-3-3 universal signaling regulator. Structure.

[CR98] Snoeckx LH, Cornelussen RN, Van Nieuwenhoven FA, Reneman RS, Van Der Vusse GJ (2001). Heat shock proteins and cardiovascular pathophysiology. Physiol Rev.

[CR99] Sontag EM, Samant RS, Frydman J (2017). Mechanisms and functions of spatial protein quality control. Annu Rev Biochem.

[CR100] Strauch A, Haslbeck M (2016). The function of small heat-shock proteins and their implication in proteostasis. Proteostasis.

[CR101] Sugiyama Y, Suzuki A, Kishikawa M, Akutsu R, Hirose T, Waye MM, Tsui SK, Yoshida S, Ohno S (2000). Muscle develops a specific form of small heat shock protein complex composed of MKBP/HSPB2 and HSPB3 during myogenic differentiation. J Biol Chem.

[CR102] Sutherland-Smith AJ (2011). Filamin structure, function and mechanics: are altered filamin-mediated force responses associated with human disease?. Biophys Rev.

[CR103] Tessier DJ, Komalavilas P, Panitch A, Joshi L, Brophy CM (2003). The small heat shock protein (HSP) 20 is dynamically associated with the actin cross-linking protein actinin. J Surg Res.

[CR104] Treweek TM, Meehan S, Ecroyd H, Carver JA (2015). Small heat-shock proteins: important players in regulating cellular proteostasis. Cell Mol Life Sci.

[CR105] Ulbricht A, Eppler FJ, Tapia VE, van der Ven PF, Hampe N, Hersch N, Vakeel P, Stadel D, Haas A, Saftig P, Behrends C, Furst DO, Volkmer R, Hoffmann B, Kolanus W, Hohfeld J (2013). Cellular mechanotransduction relies on tension-induced and chaperone-assisted autophagy. Curr Biol.

[CR106] Unger A, Beckendorf L, Bohme P, Kley R, von Frieling-Salewsky M, Lochmuller H, Schroder R, Furst DO, Vorgerd M, Linke WA (2017). Translocation of molecular chaperones to the titin springs is common in skeletal myopathy patients and affects sarcomere function. Acta Neuropathol Commun.

[CR107] van der Ven PF, Ehler E, Vakeel P, Eulitz S, Schenk JA, Milting H, Micheel B, Furst DO (2006). Unusual splicing events result in distinct Xin isoforms that associate differentially with filamin c and Mena/VASP. Exp Cell Res.

[CR108] van der Ven PF, Obermann WM, Lemke B, Gautel M, Weber K, Furst DO (2000). Characterization of muscle filamin isoforms suggests a possible role of gamma-filamin/ABP-L in sarcomeric Z-disc formation. Cell Motil Cytoskeleton.

[CR109] Vos MJ, Kanon B, Kampinga HH (2009). HSPB7 is a SC35 speckle resident small heat shock protein. Biochim Biophys Acta.

[CR110] Waters ER (2013). The evolution, function, structure, and expression of the plant sHSPs. J Exp Bot.

[CR111] Weeks SD, Baranova EV, Heirbaut M, Beelen S, Shkumatov AV, Gusev NB, Strelkov SV (2014). Molecular structure and dynamics of the dimeric human small heat shock protein HSPB6. J Struct Biol.

[CR112] Weeks SD, Muranova LK, Heirbaut M, Beelen S, Strelkov SV, Gusev NB (2018). Characterization of human small heat shock protein HSPB1 alpha-crystallin domain localized mutants associated with hereditary motor neuron diseases. Sci Rep.

[CR113] Wettstein G, Bellaye PS, Micheau O, Bonniaud P (2012). Small heat shock proteins and the cytoskeleton: an essential interplay for cell integrity?. Int J Biochem Cell Biol.

[CR114] Wu T, Mu Y, Bogomolovas J, Fang X, Veevers J, Nowak RB, Pappas CT, Gregorio CC, Evans SM, Fowler VM, Chen J (2017). HSPB7 is indispensable for heart development by modulating actin filament assembly. Proc Natl Acad Sci U S A.

[CR115] Yin B, Tang S, Xu J, Sun J, Zhang X, Li Y, Bao E (2019). CRYAB protects cardiomyocytes against heat stress by preventing caspase-mediated apoptosis and reducing F-actin aggregation. Cell Stress Chaperones.

[CR116] Zhu Y, Bogomolovas J, Labeit S, Granzier H (2009). Single molecule force spectroscopy of the cardiac titin N2B element: effects of the molecular chaperone alphaB-crystallin with disease-causing mutations. J Biol Chem.

